# Glycolysis Is Dynamic and Relates Closely to Respiration Rate in Stored Sugarbeet Roots

**DOI:** 10.3389/fpls.2017.00861

**Published:** 2017-05-24

**Authors:** Clarice A. Megguer, Karen K. Fugate, Abbas M. Lafta, Jocleita P. Ferrareze, Edward L. Deckard, Larry G. Campbell, Edward C. Lulai, Fernando L. Finger

**Affiliations:** ^1^Departamento de Biologia Vegetal, Universidade Federal de ViçosaViçosa, Brazil; ^2^Northern Crop Science Laboratory, Agricultural Research Service (USDA), FargoND, United States; ^3^Department of Plant Sciences, North Dakota State University, FargoND, United States; ^4^Departamento de Fitotecnia, Universidade Federal de ViçosaViçosa, Brazil

**Keywords:** *Beta vulgaris*, phosphofructokinase, postharvest, pyruvate kinase, storage

## Abstract

Although respiration is the principal cause of the loss of sucrose in postharvest sugarbeet (*Beta vulgaris* L.), the internal mechanisms that control root respiration rate are unknown. Available evidence, however, indicates that respiration rate is likely to be controlled by the availability of respiratory substrates, and glycolysis has a central role in generating these substrates. To determine glycolytic changes that occur in sugarbeet roots after harvest and to elucidate relationships between glycolysis and respiration, sugarbeet roots were stored for up to 60 days, during which activities of glycolytic enzymes and concentrations of glycolytic substrates, intermediates, cofactors, and products were determined. Respiration rate was also determined, and relationships between respiration rate and glycolytic enzymes and metabolites were evaluated. Glycolysis was highly variable during storage, with 10 of 14 glycolytic activities and 14 of 17 glycolytic metabolites significantly altered during storage. Changes in glycolytic enzyme activities and metabolites occurred throughout the 60 day storage period, but were greatest in the first 4 days after harvest. Positive relationships between changes in glycolytic enzyme activities and root respiration rate were abundant, with 10 of 14 enzyme activities elevated when root respiration was elevated and 9 glycolytic activities static during periods of unchanging respiration rate. Major roles for pyruvate kinase and phosphofructokinase in the regulation of postharvest sugarbeet root glycolysis were indicated based on changes in enzymatic activities and concentrations of their substrates and products. Additionally, a strong positive relationship between respiration rate and pyruvate kinase activity was found indicating that downstream TCA cycle enzymes were unlikely to regulate or restrict root respiration in a major way. Overall, these results establish that glycolysis is not static during sugarbeet root storage and that changes in glycolysis are closely related to changes in sugarbeet root respiration.

## Introduction

After harvest, sugarbeet (*Beta vulgaris* L.) roots are stored in large outdoor piles for up to 200 days before they are processed or frozen for long-term storage (Campbell and Klotz, 2006). During this time, root sucrose content declines. Under ideal storage conditions, sugarbeet roots stored for 100 days lost 3–9% of the sugar present at harvest (Beaudry et al., 2011). In commercial practice, ideal storage conditions are seldom achieved, and losses of 50% or more of the sucrose present at harvest have occurred in roots stored under unfavorable environmental conditions (Kenter and Hoffmann, 2009). Postharvest diseases and conversion of sucrose to other carbohydrates such as glucose, fructose, and raffinose contribute to postharvest sucrose losses. Root respiration, however, is typically the principal cause of storage sucrose loss, with estimates that up to 80% of the sucrose lost in storage is due to this process (Vukov and Hangyál, 1985).

Respiration is the oxidative process that converts cellular organic compounds to carbon dioxide and water to generate metabolic energy in the form of ATP. In non-photosynthetic organs, such as harvested sugarbeet roots, respiration is the primary source of metabolic energy and generates the ATP and carbon compounds that are needed to maintain metabolism, fuel defense mechanisms against storage pathogens, and heal wounds received during harvest and piling operations. Carbohydrates, amino acids, proteins, organic acids, and lipids can serve as respiratory substrates in plants. In sugarbeet roots, however, nearly all respiration occurs by catabolism of sucrose and is carried out by the sequential activities of sucrolytic, glycolytic, TCA cycle, and electron transport chain pathways (Barbour and Wang, 1961; Wang and Barbour, 1961). Starch, a common substrate for respiration in storage organs, is undetectable in sugarbeet root (Turesson et al., 2014).

Respiration in plants is regulated by three possible mechanisms: (1) a restriction in the availability of respiratory substrates, (2) a limitation in respiratory capacity – due to the combined capacities of the electron transport chain’s terminal oxidases (cytochrome *c* oxidase and alternative oxidase), or (3) cellular energy charge, often expressed as the ratio of ATP to ADP (Dwivedi, 2000; Covey-Crump et al., 2002). In harvested sugarbeet roots, respiratory capacity greatly exceeds root respiration rate (Klotz et al., 2008), suggesting that respiratory capacity does not limit respiration rate. Respiration rate is also unrelated to and unaffected by ADP concentration, ATP concentration, or the ratio of ATP to ADP (Klotz et al., 2008), indicating that cellular energy charge does not control root respiration rate. Therefore, sugarbeet root respiration, by default, is likely to be regulated by the availability of respiratory substrates.

The substrates limiting sugarbeet root respiration are presently unknown, but may include any substrate or cofactor in the pathways involved in the oxidation of sucrose to carbon dioxide. The high concentration of sucrose in the sugarbeet taproot suggests that sucrose is unlikely to be a limiting substrate. The activities of sucrolytic enzymes are also unlikely to restrict respiration rate since these enzymes are abundant and their activities are not correlated with root respiration rate (Klotz et al., 2006, 2008). While a few studies have found that restricting enzymes in the TCA cycle can limit respiration rate (Araújo et al., 2008; van der Merwe et al., 2010), the concentration of TCA cycle intermediates and the enzymes that generate them have generally been found to be unrelated to respiration rate (Lin et al., 2004; Araújo et al., 2012 and references therein). In contrast, glycolytic enzymes, intermediates, cofactors, or products have been identified as restrictors of respiration rate in a number of plant species (Beaudry et al., 1989; Hatzfeld and Stitt, 1991; Zabalza et al., 2009).

Little information is available regarding glycolytic enzymes, substrates, intermediates, or products in postharvest sugarbeet roots. To date, the activities for four glycolytic enzymes [hexokinase (HK), fructokinase (FK), phosphofructokinase (PFK), and pyruvate kinase (PK)] and two glycolytic substrates (glucose and fructose) have been determined in response to wounding in the 14 days following harvest (Klotz et al., 2006); the concentrations of glycolytic substrates, intermediates, and product concentrations have been determined in response to wounding in the first 3 days after harvest (DAH) (Lafta and Fugate, 2011); and the activities of three glycolytic enzymes [HK, FK, and glucose 6-phosphate isomerase (G6PI)] have been determined in response to unusually high temperatures in the 10 days following harvest (Sakalo and Tyltu, 1997). Information that is limited to the first 2 weeks after harvest, therefore, is available for 5 of the 14 enzymes that contribute to glycolysis in postharvest sugarbeet roots; no information is available for the other 9 enzyme activities that participate in glycolysis. Moreover, no information is available regarding changes in glycolytic enzyme activities or the concentrations of glycolytic intermediates or products under the prolonged, low-temperature conditions typical of sugarbeet storage.

Because of the fundamental importance of glycolysis to postharvest sugarbeet root metabolism, the potential of glycolysis to regulate storage respiration rate, and the general lack of information regarding glycolysis in stored sugarbeet roots, research was carried out to characterize sugarbeet root glycolysis at harvest and during storage, using temperature and humidity conditions considered ideal for sugarbeet root storage. The activities of glycolytic enzymes and the concentrations of glycolytic substrates, intermediates, cofactors, and products were determined at harvest and throughout 60 days in storage. Root respiration rate was also determined and relationships between respiration rate, enzyme activities, and glycolytic metabolite concentrations were determined. The purpose of this research was to gain a basic understanding of glycolysis in harvested sugarbeet roots and determine how it changes during storage, especially in relation to root respiration rate.

## Materials and Methods

### Plant Material, Growing Conditions, and Storage Conditions

Sugarbeet plants of hybrid VDH66156 (SES VanderHave, Tienen, Belgium) were greenhouse-grown in Sunshine Mix #1 (Sun Gro Horticulture, Vancouver, BC, Canada) in 15 L pots. Plants were watered as needed, fertilized with a controlled release fertilizer (Multicote 4, Sun Gro Horticulture), and provided supplemental light under a 16 h light/8 h dark regime throughout the growing period. Roots were harvested 16 weeks after planting, leaves, and vegetative buds were removed, and roots were washed to remove potting medium. Roots ranged in size from 0.5 to 0.8 kg and averaged 0.61 kg. Roots were stored at 10°C and 90 ± 5% relative humidity (RH) for 10 days, then incubated at 4°C and 90 ± 5% RH for up to 60 days. After 0, 1, 2, 3, 4, 7, 10, 30, and 60 days in storage, seven randomly selected roots were removed from storage, and roots were destructively sampled by collecting a longitudinal quarter section from each root. Root sections were flash frozen in liquid nitrogen, lyophilized, ground to a fine powder, and stored at -80°C until analysis. Root respiration rate was determined after 1, 2, 3, 4, 7, 10, 30, and 60 days storage. Respiration rate was determined using the same seven roots as described above and was determined using the whole roots prior to the collection of tissue samples. Day 0 respiration rate determinations were not made since root temperatures were >20°C and could not be compared with subsequent respiration determinations that were made at 10°C. For all analyses, individual roots were the experimental unit with seven replicate roots per time point. Experiment was conducted twice.

### Respiration Rate

Respiration rates of individual roots were determined at 10°C by infrared CO_2_ analysis using a LICOR 6400 gas analyzer (Lincoln, NE, United States) attached to a 7 L sample chamber as previously described (Haagenson et al., 2006). For respiration rate determinations at 30 and 60 days of storage, roots were equilibrated at 10°C for 2 days prior to measurement. Respiration rate was expressed as μmol CO_2_ kg fresh weight^-1^ h^-1^.

### Soluble Protein Extraction and Quantification

Soluble protein extracts were prepared by adding 10 volumes (v/w) of an extraction buffer to lyophilized tissue. For phosphoenolpyruvate phosphatase (PEPase) activity assays, extraction buffer contained 100 mM HEPES-NaOH, pH 7.5, 5 mM dithiothreitol (DTT), 1 mM EDTA, 0.5% Triton X-100, and 0.5% bovine serum albumin (BSA) (Lu et al., 2014). For all other assays, the extraction buffer contained 100 mM HEPES-NaOH, pH 7.5, 2 mM MgCl_2_, 5 mM DTT, and 20 mM Na_2_SO_3_. The resulting suspensions were vortexed for 30 s, sonicated for 10 min using a Mettler Electronics Model 4.6 sonicator (Anaheim, CA, United States) operating at 67 KHz, and centrifuged at 17,000 × *g* for 20 min. All operations were conducted at 4°C. Supernatants were desalted using a Sephadex G-25 (GE Healthcare Bio-Sciences AB, Uppsala, Sweden) column pre-equilibrated with 10 mM HEPES-NaOH, pH 7.2. Total protein concentration was determined with a Bio-Rad Protein Assay Kit (Hercules, CA, United States) using BSA as standard.

### Enzyme Activity Assays

Enzyme activities were determined using modifications of the protocols of Moorhead and Plaxton (1988) for HK, FK, PFK, and PK, the protocols of Burrell et al. (1994) for G6PI, aldolase (ALD), triose phosphate isomerase (TPI), phosphoglycerate mutase (PGlyM), phosphoglycerate kinase (PGK), enolase (ENO), pyrophosphate-dependent phosphofructokinase (PFP), and UDP-glucose pyrophosphorylase (UDPase), the protocol of Plaxton (1990) for glyceraldehyde 3-phosphate isomerase (GAPDH), the protocol of Lu et al. (2014) for PEPase, and the protocols of Manjunath et al. (1998) and Davies et al. (2003) for phosphoglucomutase (PGM). Assays were performed at 25°C and coupled enzyme activity with NADH or NAD^+^ formation. NADH formation or consumption was measured at 340 nm using a SpectraMAX Plus microplate spectrophotometer (Molecular Devices Corp., Sunnyvale, CA, United States). Assay components were as follows. HK: 125 mM HEPES-NaOH (pH 7.5), 10 mM MgCl_2_, 7 mM glucose, 1.5 mM NAD^+^, 2 U mL^-1^ glucose 6-phosphate dehydrogenase (G6P-DH), and 0.25 mM ATP; FK: 125 mM HEPES-NaOH (pH 7.5), 10 mM MgCl_2_, 3 mM fructose, 1.5 mM NAD^+^, 2 U mL^-1^ G6P-DH, 6 U mL^-1^ phosphoglucose isomerase, and 0.25 mM ATP; PFK: 50 mM Tris-HCl (pH 8.0), 5 mM MgCl_2_, 2 mM EDTA, 2 mM fructose 6-phosphate, 0.1 mM NADH, 2 U mL^-1^ ALD, 2 U mL^-1^ TPI, 5 U mL^-1^ glycerol 3-phosphate dehydrogenase, and 0.12 mM ATP; PK: 50 mM HEPES-NaOH (pH 7.0), 50 mM KCl, 10 mM MgCl_2_, 2 mM DTT, 0.4 mg mL^-1^ BSA, 1 mM phosphoenolpyruvate, 0.075 mM NADH, 26 U mL^-1^ lactate dehydrogenase, and 1 mM ADP; PGM: 50 mM Tris-HCl (pH 7.5), 10 mM MgCl_2_, 30 μM glucose 1,6-bisphosphate, 0.5 mM NAD^+^, 2 U mL^-1^ G6P-DH, and 0.9 mM glucose 1-phosphate (G1P); G6PI: 75 mM glycyl-glycine (pH 8.5), 10 mM MgCl_2_, 1 mM NAD^+^, 1 mM fructose 6-phosphate, 0.5 U mL^-1^ G6P-DH; ALD: 40 mM HEPES-NaOH (pH 7.7), 0.1 mM NADH, 5 mM fructose 1,6-bisphosphate (F1,6P), 1.7 U mL^-1^ glycerol 3-phosphate dehydrogenase, 17 U mL^-1^ TPI; TPI: 100 mM HEPES-NaOH (pH 8.0), 5 mM EDTA, 0.2 mM NADH, 1.5 mM DL-glyceraldehyde 3-phosphate, and 1 U mL^-1^ glycerol 3-phosphate dehydrogenase; PGK: 100 mM HEPES-NaOH (pH 7.6), 1 mM EDTA, 2 mM MgSO_4_, 0.2 mM NADH, 6.5 mM 3-phosphoglycerate, 1 mM ATP, and 3.3 U mL^-1^ glycerol 3-phosphate dehydrogenase; PGlyM: 100 mM Tris-HCl (pH 7.6), 10 mM MgSO_4_, 2.7 mM ADP, 0.2 mM NADH, 3 mM 3-phosphoglycerate, 1 U mL^-1^ ENO, 5 U mL^-1^ PK, 6 U mL^-1^ lactate dehydrogenase, and 50 mM 3-phosphoglycerate; ENO: 100 mM HEPES-NaOH (pH 7.5), 10 mM MgCl_2_, 1 mM NADH, 2.7 mM ADP, 0.5 mM 2-phosphoglycerate, 5 U mL^-1^ PK, 6 U mL^-1^ lactate dehydrogenase, and 10 mM 2-phosphoglycerate; PEPase: 50 mM Tris-HCl (pH 7.5), 1 mM phosphoenolpyruvate, 4 mM MgCl_2_, 0.2 mM NADH, and 3 U lactate dehydrogenase; PFP: 100 mM Tris-HCl (pH 8.0), 5 mM fructose 6-phosphate, 2 mM sodium pyrophosphate, 5 mM MgCl_2_, 0.20 mM NADH, 1 U mL^-1^ ALD, 1.3 U mL^-1^ glycerol 3-phosphate dehydrogenase, and 10 U mL^-1^ TPI; UDPase: 100 mM Tris-HCl (pH 8.0), 5 mM MgCl_2_, 1.6 mM NAD^+^, 0.8 mM UDP-glucose (UDPG), 4 U mL^-1^ PGM, 4 U mL^-1^ G6P-DH, and 0.4 mM sodium pyrophosphate; GAPDH: 100 mM Tris-HCl (pH 7.8), 4.5 mM 3-phosphoglycerate, 8 mM MgSO_4_, 0.32 mM NADH, 2 mM ATP, 1 mM EDTA, 2 mM DTT, and 1.8 U mL^-1^ PGK. Reactions were initiated by addition of ATP for HK, FK, and PFK assays; ADP for PK assay; G1P for PGM assay; 3-phosphoglycerate for PGlyM assay; 2-phosphoglycerate for ENO assay; sodium pyrophosphate for UDPase assay; and protein extract for G6PI, ALD, TPI, PGK, PEPase, PFP, and GAPDH assays. Additional details for enzyme assay protocols are available in Supplementary Table S1.

### Metabolite Extraction and Quantification

Metabolites were extracted from lyophilized tissues as previously described using 80% aqueous MeOH for carbohydrate, organic acid, and nucleotide extractions, 0.1 M HCl for NAD^+^ extraction, and 0.1 M NaOH for NADH extraction (Lafta and Fugate, 2011). Sucrose, glucose, fructose, fructose 6-phosphate, glucose 6-phosphate (G6P), G1P, F1,6P, triose phosphates, NAD^+^, and NADH were quantified using previously described enzyme-coupled endpoint spectroscopic assays (Spackman and Cobb, 2001; Lafta and Fugate, 2011). Phosphoenolpyruvate, pyruvate, UDPG, UDP, UTP, ADP, and ATP were quantified by HPLC as described (Lafta and Fugate, 2011). All metabolite concentrations are expressed per g dry weight of tissue. Additional details for metabolite analyses are available in Supplementary Table S2.

### Statistical Analysis

Statistical analyses were conducted with Minitab Statistical Software (ver. 16.2.3, State College, PA, United States). Analysis of variance (ANOVA) and Fisher’s protected least significant difference (LSD) tests were used to determine significant differences between time points. LSDs were determined only when the overall F-ratio from the ANOVA was statistically significant. Relationships between data were analyzed using Pearson product-moment correlation and principal component (PCA) analyses. PCA was conducted after standardization of data. For all analyses, α = 0.05.

## Results

### Root Respiration

**FIGURE 1:**
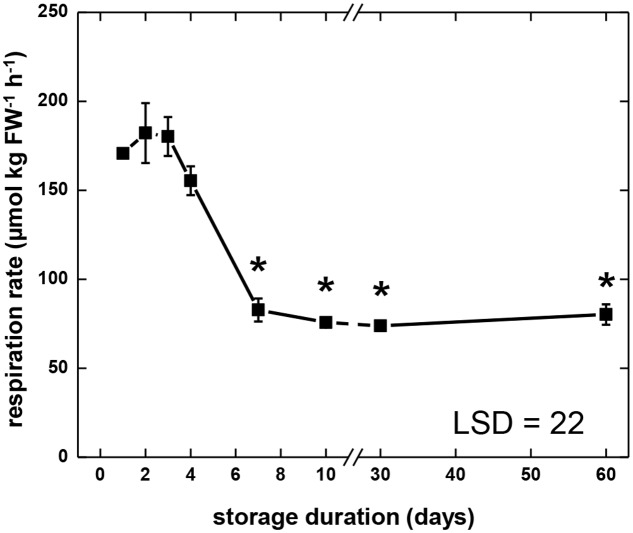
**Respiration rate of sugarbeet roots during 60 days of storage**. Freshly harvested roots were stored for 10 days at 10°C and 90 ± 5% relative humidity (RH), then stored at 4°C, 90 ± 5% RH for 50 days. Respiration rate of individual roots was measured as CO_2_ produced at 10°C per kg fresh weight per hour. For measurements at 30 and 60 days, roots were equilibrated for 2 days at 10°C prior to measurement. Error bars are ±1 standard error of the mean, where *n* = 7. Asterisks denote data that are significantly different from the initial respiration rate, with α = 0.05.

During 60 days storage, sugarbeet root respiration rate exhibited three distinct stages (**Figure 1**). Respiration rate was relatively high and constant 1–4 DAH, declined 4–7 DAH, and was relatively low and constant 7–60 DAH. During the first stage (1–4 DAH), roots respired at an average respiration rate of 172 μmol CO_2_ kg fresh weight^-1^ h^-1^. In the second stage (4–7 DAH) respiration rate declined by more than 50% relative to the average respiration rate for days 1–4. Throughout the third stage (7–60 DAH), root respiration was relatively constant and averaged 78 μmol CO_2_ kg fresh weight^-1^ h^-1^.

### Glycolytic Enzyme Activities

**FIGURE 2:**
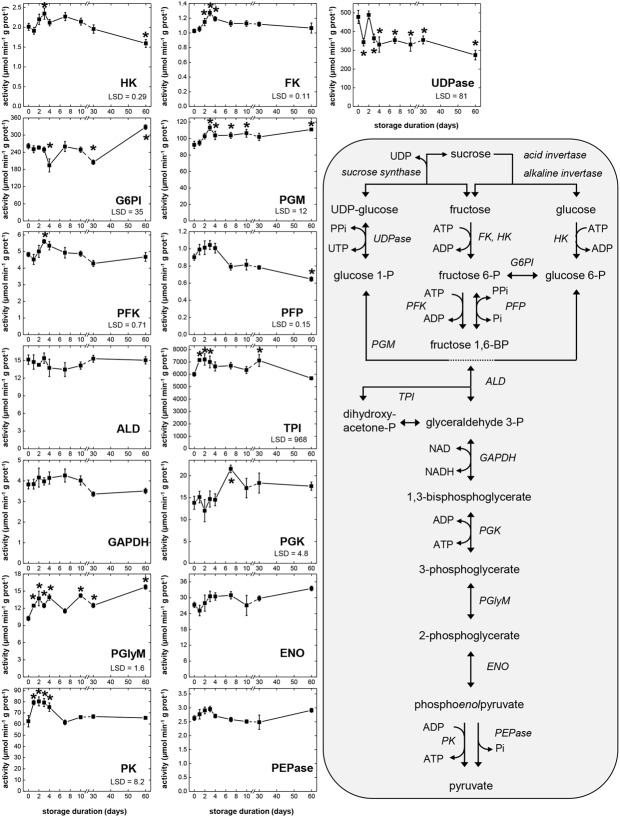
**Glycolytic enzyme activities in sugarbeet roots during 60 days storage and schematic of glycolytic reactions**. Roots were stored for 10 days at 10°C and 90 ± 5% RH, then stored at 4°C, 90 ± 5% RH for 50 days. All activities are expressed as μmol min^-1^ g^-1^ protein. Abbreviations for enzymes are provided in **Table 1**. Error bars are ±1 standard error of the mean, where *n* = 7. Fisher’s least significant differences (LSDs) are provided in lower left corner of graphs with α = 0.05. Asterisks denote data that are significantly different from that at 0 days.

Activities of 14 glycolytic enzymes and UDP-glucose pyrophosphorylase (UDPase) were determined during storage (**Figure 2**). Activity assays were carried out at optimum pH and substrate conditions and the rates measured were indicative of enzymatic capacity or the concentration of active enzyme present. The range of activities observed for each enzyme during storage and the abbreviations used to describe enzymes are provided in **Table 1**. Large differences in the magnitude of glycolytic enzymes and UDPase activities were observed (**Table 1**). Overall, a 7800-fold difference in activity was observed between TPI, the most active enzyme in this study, and PFP, the enzyme exhibiting the lowest activity. Other enzymes exhibiting high activity with mean activity greater than 100 μmol min^-1^ g^-1^, included UDPase, G6PI, and PGM. Enzymes with low activities, with a mean activity of 3 μmol min^-1^ g^-1^ or lower, included phosphoenolpyruvate phosphatase (PEPase), HK, and FK, in addition to PFP.

**Table 1:** Enzyme Commission (E.C.) numbers, abbreviations used, and the range of activities for glycolytic enzymes that were characterized in sugarbeet roots during 60 days storage.

Enzyme	E.C.	Abbreviation	Activity (μmol min^-1^ g^-1^)
Triose phosphate isomerase	5.3.1.1	TPI	5680 – 7190
UDP-glucose pyrophosphorylase	2.7.7.9	UDPase	275 – 489
Glucose 6-phosphate isomerase	5.3.1.9	G6PI	194 – 328
Phosphoglucomutase	5.4.2.2	PGM	92 – 113
Pyruvate kinase	2.7.1.40	PK	62 – 80
Enolase	4.2.1.11	ENO	25 – 34
Phosphoglycerate kinase	2.7.2.3	PGK	12 – 22
Aldolase	4.1.2.13	ALD	13 – 15
Phosphoglycerate mutase	2.7.5.3	PGlyM	10 – 16
ATP-dependent phosphofructokinase	2.1.7.11	PFK	4.3 – 5.6
Glyceraldehyde 3-phosphate dehydrogenase	1.2.1.12	GAPDH	3.4 – 4.3
Phosphoenolpyruvate phosphatase	3.1.3.60	PEPase	2.5 – 3.0
Hexokinase	2.7.1.1	HK	1.6 – 2.3
Fructokinase	2.7.1.4	FK	1.0 – 1.3
Pyrophosphate-dependent phosphofructokinase	2.7.1.90	PFP	0.65 – 1.0

*Activities are presented as the range of activities that occurred during 60 days storage (lowest observed activity to highest observed activity), using the mean activities from samples at 0, 1, 2, 3, 4, 7, 10, 30, and 60 days after harvest. Enzymes are listed in order of decreasing mean activity, with activity expressed as the quantity of substrate consumed min^-*1*^ g^-*1*^ protein. Roots were stored 10 days at 10°C and 90 ± 5% relative humidity (RH), then stored at 4°C, 90 ± 5% RH for 50 days*.

For the 15 enzymatic activities determined, all but four (ALD, GAPDH, ENO, and PEPase) changed significantly during storage (**Figure 2**). Seven glycolytic enzymes exhibited elevated activity during the first 4 days in storage, when root respiration rate was high. These enzymes were HK, FK, PGM, PFK, TPI, PGlyM, and PK. Three other enzymes, PFP, ENO, and PEPase, also were elevated during the first 4 days in storage, although these elevations were not statistically significant. Most of the enzymes exhibiting elevated activity during the first 4 days in storage (HK, FK, PGM, PFK, PFP, and PEPase) reached their maximum activity 3 days after harvest. Three enzymes (TPI, PGlyM, and PK), however, were elevated throughout days 1–4. After 4 days in storage, activity declined for four of the enzymes (FK, PFK, PFP, and PK) that were elevated during the first 4 days in storage, coincidental with a decline in root respiration rate (**Figure 1**). A decline in activity for PEPase after 4 days in storage was also observed, although its decline was not significant and transient. Between 7 and 60 days after harvest, when root respiration was relatively unchanged, activities of nine enzymes (FK, PGM, PFK, ALD, GAPDH, PGK, ENO, PK, and PEPase) were unchanged. However, while root respiration rate was greatly reduced during this period, activities for two of these enzymes (PGM and PGK) were elevated.

**FIGURE 3:**
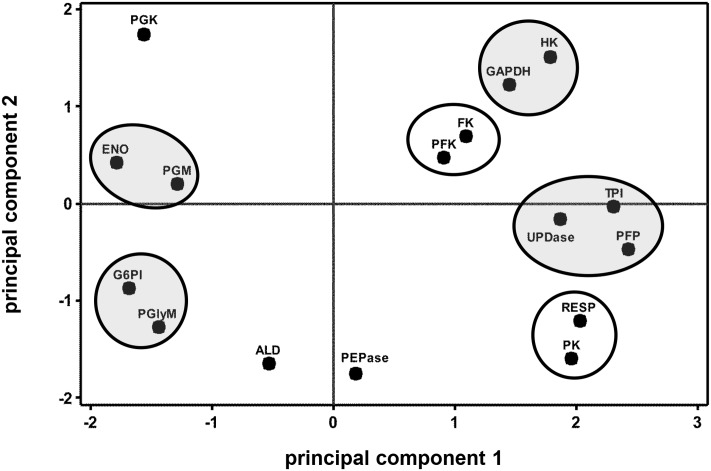
**Principal component analysis (PCA) score plot of the changes in glycolytic enzyme activities and respiration rate (RESP) during 60 days storage**. Ovals identify groups of metabolites that exhibit similarities in storage-related concentration changes. Ovals are gray where metabolites share similar changes in concentration, but have no obvious connection with each other. Roots were stored 10 days at 10°C and 90 ± 5% RH, then stored at 4°C, 90 ± 5% RH for an additional 50 days. Principal components 1 and 2 account for 41 and 21%, respectively, of the variance in the data after standardization. Abbreviations used for enzymes are defined in **Table 1**. PCA loading plot is available in Supplementary Figure S1.

Correlation analysis of storage-related changes in glycolytic enzyme activities and respiration rate revealed numerous relationships among enzyme activities and between enzyme activities and respiration rate (**Table 2**). Included in these were positive correlations between three ATP-dependent, early glycolytic enzymes (i.e., FK with HK and PFK), positive correlations between respiration rate with PFP and PK activities, and a negative correlation of respiration rate and PGK activity. Principal component analysis was also used to identify relationships between storage-related changes in glycolytic enzyme activities and respiration rate (**Figure 3**). PCA confirmed similarities in storage-related changes between FK and PFK and between respiration rate and PK activity.

**Table 2:** Significant correlations between glycolytic enzyme activities and respiration rate (RESP) in roots during 60 days storage (α = 0.05).

Enzyme	Positive correlations	Negative correlations
HK	FK, GAPDH	–
FK	HK, PFK	–
UDPase	TPI	–
G6PI	–	–
PGM	ENO	–
PFK	FK	–
PFP	TPI, PK, RESP	PGK
ALD	–	GAPDH
TPI	UDPase, PFP	PGlyM
GAPDH	HK	ALD
PGK	–	PFP, PK, RESP
PGlyM	–	TPI
ENO	PGM	–
PK	PFP, RESP	PGK
PEPase	–	–
RESP	PFP, PK	PGK

*Roots were stored 10 days at 10°C and 90 ± 5% RH, then stored at 4°C, 90 ± 5% RH for 50 days. Abbreviations used for enzymes are defined in **Table 1**. – is used to denote the absence of any significant correlations. Correlation coefficients and *P*-values are available in Supplementary Table S3*.

### Glycolytic Metabolite Concentrations

**FIGURE 4:**
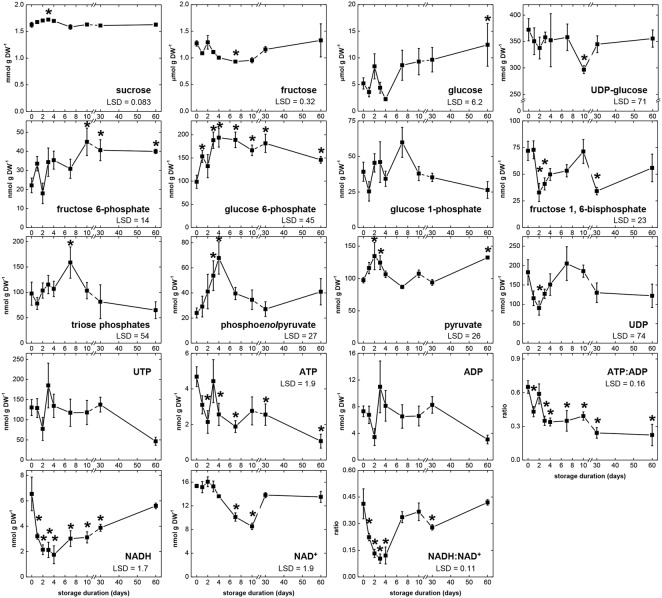
**Concentrations of glycolytic pathway metabolites, ATP:ADP ratio, and NADH:NAD^+^ ratio during 60 days storage**. Roots were stored for 10 days at 10°C and 90 ± 5% RH, then stored at 4°C, 90 ± 5% RH for 50 days. Concentrations are expressed per gram dry weight. ATP:ADP ratio is provided as a measure of cellular energy status. NADH:NAD^+^ ratio is provided as a measure of cellular redox status. Error bars are ±1 standard error of the mean, where *n* = 7. Fisher’s LSDs are provided in lower left corner of graphs with α = 0.05. Asterisks denote data that are significantly different from that at 0 days.

Changes in concentrations of 17 metabolites that are substrates, intermediates, products, or cofactors of glycolysis were determined during 60 days storage (**Figure 4**), with the abbreviations used for these metabolites listed in **Table 3**. Changes in the ratios of ATP:ADP and NADH:NAD^+^ were also determined as indicators of cellular energy charge and cellular redox status, respectively. For 14 of 17 metabolites, significant concentration changes occurred during storage. Only G1P, UTP, and ADP exhibited no significant changes during storage. Four metabolites (sucrose, G6P, phosphoenolpyruvate [PEP], and pyruvate [Pyr]) were generally elevated during the first 4 DAH, when respiration rate was high. During this same period, concentrations of F1,6P, UDP, ATP, and NADH generally declined, indicating catabolism of these compounds exceeded their biosynthesis. Also declining 1–4 DAH were the ratios of ATP:ADP and NADH:NAD^+^, suggesting a reduction in cellular energy status and redox status during the period of elevated respiration. Between 4 and 7 DAH, significant decreases in three metabolites (sucrose, PEP, and NAD^+^) and significant increases in glucose concentration and the ratio of NADH:NAD^+^ occurred coincidental with a decrease in root respiration rate. From 7 to 60 DAH, when root respiration was relatively constant, the concentrations of nine metabolites were statistically unchanged. These metabolites were sucrose, glucose, UDPG, G6P, G1P, PEP, UTP, ATP, and ADP. While respiration rate was greatly reduced during this time from the high rates observed 1–4 DAH, concentrations of glucose and G6P were generally elevated and concentrations of UDPG, G1P, UTP, ATP, and ADP were generally similar to those observed between 1 and 4 DAH. Only sucrose and PEP concentrations were statistically constant and reduced from their concentrations at 1–4 DAH.

**Table 3:** Abbreviations used for glycolytic intermediates characterized during 60 days storage.

Metabolite	Abbreviation
Fructose 6-phosphate	F6P
Glucose 6-phosphate	G6P
Glucose 1-phosphate	G1P
Fructose 1,6-bisphosphate	F1,6P
Triose phosphates	trioseP
Phosphoenolpyruvate	PEP
Pyruvate	Pyr
Uridine diphosphate glucose	UDPG

**FIGURE 5:**
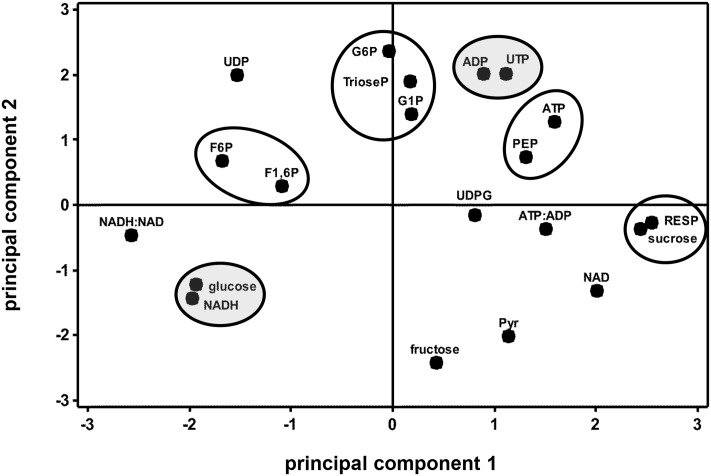
**Principal component analysis score plot of the changes in glycolytic pathway metabolite concentrations, ATP:ADP ratio, NADH:NAD^+^ ratio, and respiration rate (RESP) during 60 days storage**. Ovals identify groups of metabolites that exhibit similarities in storage-related concentration changes. Ovals are gray where metabolites share similar changes in concentration, but have no obvious connection with each other. Roots were stored 10 days at 10°C and 90 ± 5% RH, then stored at 4°C, 90 ± 5% RH for 50 days. Principal components 1 and 2 account for 34 and 30%, respectively, of the variance in the data after standardization. Abbreviations used for metabolites are defined in **Table 3**. PCA loading plot is available in Supplementary Figure S2.

Correlation analysis of storage-related changes in metabolite concentrations, ATP:ADP ratio, NADH:NAD^+^ ratio, and respiration rate revealed numerous positive and negative correlations between metabolites, cellular energy charge, cellular redox status, and respiration rate (**Table 4**). Among these relationships, root respiration rate was positively correlated with sucrose and NAD^+^ concentrations and negatively correlated with glucose concentration and cellular redox status, as measured by the NADH:NAD^+^ ratio. A negative correlation between cellular energy charge, as measured by the ATP:ADP ratio, and F6P was also observed. The similarity in storage-related changes in respiration rate and sucrose was confirmed by principal component analysis (**Figure 5**). PCA also revealed similarities in concentration changes between F6P and F1,6P, between PEP and ATP, and between G6P, G1P, and triose phosphates.

**Table 4:** Significant correlations between glycolytic pathway metabolites, ATP:ADP ratio, NADH:NAD^+^ ratio, and respiration rate (RESP) in roots during 60 days storage.

Metabolite	Positive correlations	Negative correlations
Sucrose	RESP	NADH:NAD^+^
Fructose	Pyr	UDP
Glucose	NADH, NADH:NAD^+^	RESP
UDPG	–	–
F6P	–	ATP:ADP
G6P	ADP, UTP	Pyr
G1P	trioseP	–
F1,6P	–	–
trioseP	G1P	–
PEP	–	–
Pyr	Fructose	G6P, UDP
UDP	–	Fructose, Pyr, NAD^+^
UTP	G6P, ADP, ATP	–
ADP	G6P, UTP, ATP	–
ATP	UTP, ADP	–
ATP:ADP	–	F6P
NADH	Glucose, NADH:NAD^+^	–
NAD^+^	RESP	UDP
NADH:NAD^+^	Glucose, NADH	Sucrose, RESP
RESP	Sucrose, NAD^+^	Glucose, NADH:NAD^+^

*Roots were stored 10 days at 10°C and 90 ± 5% RH, then stored at 4°C, 90 ± 5% RH for an additional 50 days. Abbreviations used for metabolites are defined in **Table 3**. ATP:ADP ratio is used as a measure of cellular energy status. NADH:NAD^+^ ratio is used as a measure of cellular redox status. – is used to denote the absence of any significant correlations, with α = 0.05. Correlation coefficients and *P*-values are available in Supplementary Table S4*.

### Combined Analysis of Glycolytic Enzyme Activities and Metabolite Concentrations

**FIGURE 6:**
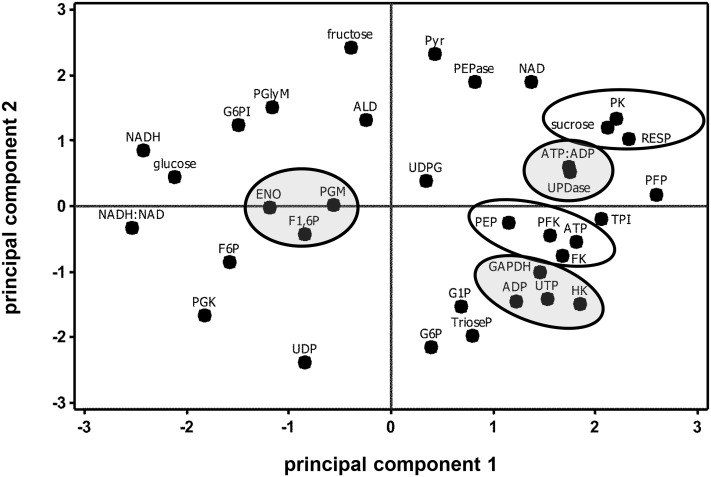
**Principal component analysis score plot of the changes in glycolytic enzyme activities, metabolite concentrations, ATP:ADP ratio, NADH:NAD^+^ ratio, and respiration rate (RESP) during 60 days storage**. Ovals identify parameters that exhibit similar storage-related changes. Ovals are gray where parameters share similar changes, but have no obvious connection with each other. Roots were stored 10 days at 10°C and 90 ± 5% RH, then stored at 4°C, 90 ± 5% RH for 50 days. Principal components 1 and 2 account for 34 and 26%, respectively, of the variance in the data after standardization. Abbreviations used for enzyme activities are defined in **Table 1**; abbreviations used for metabolites are defined in **Table 3**. PCA loading plot is available in Supplementary Figure S3.

Correlation and principal component analyses of glycolytic enzyme activities, metabolite concentrations, respiration rate, and the ratios of ATP:ADP and NADH:NAD^+^ identified additional relationships between measured parameters (**Table 5** and **Figure 6**). Among the relationships identified by correlation analysis were a positive correlation between PFK and PEP, a positive correlation between GAPDH with its substrate, trioseP, and a negative correlation between GAPDH with a product of its reaction, NADH (**Table 5**). PK was also found to be positively correlated with sucrose and NAD^+^ and negatively correlated with the ratio of NADH:NAD^+^. PCA confirmed the positive relationships between PFK and PEP and between PK and sucrose that were identified by correlation analysis (**Figure 6**). PCA also identified similar storage-related changes between PFK, FK, ATP, and PEP, and between sucrose, PK activity, and respiration rate.

**Table 5:** Significant correlations between glycolytic enzyme activities and glycolytic pathway metabolites, ATP:ADP ratio, and NADH:NAD^+^ ratio in roots during 60 days storage (α = 0.05).

Compound	Positive correlations	Negative correlations
HK	G1P, trioseP	NADH
FK	–	–
UDPase	ATP:ADP	F6P
G6PI	–	–
PGM	–	–
PFK	PEP	
PFP	Sucrose, ATP	Glucose, NADH, NADH:NAD^+^
ALD	–	–
TPI	–	–
GAPDH	trioseP	NADH
PGK	NADH:NAD^+^, UDP	Sucrose, Pyr
PGlyM	–	–
ENO	–	–
PK	Sucrose, NAD^+^	UDP, NADH:NAD^+^
PEPase	Pyr	–

*Roots were stored 10 days at 10°C and 90 ± 5% RH, then stored at 4°C, 90 ± 5% RH for 50 days. Abbreviations used for enzymes are defined in **Table 1**. Abbreviations used for metabolites are defined in **Table 3**. ATP:ADP ratio is used as a measure of cellular energy status. NADH:NAD^+^ ratio is used as a measure of cellular redox status. – is used to denote the absence of any significant correlations. Correlation coefficients and *P*-values are available in Supplementary Table S5*.

## Discussion

Determination of glycolytic enzyme activities and metabolite concentrations revealed that glycolysis is not static during sugarbeet storage, but changes with respect to storage duration. Eleven of 15 enzyme activities and 14 of 17 metabolite concentrations determined in this study changed significantly during storage (**Figures 2**, **4**). The greatest changes occurred during early storage (1–4 DAH) coincidental with high root respiration rate (**Figure 1**), the transition of the root from a sucrose-importing to an autotrophic organ, and the initiation of wound-healing processes against injuries sustained by taproot uprooting and shoot removal (Ibrahim et al., 2001). During this time, 9 of 15 enzyme activities, 7 of 17 metabolites, the ratio of ATP:ADP, and the ratio of NADH:NAD^+^ were altered relative to their values at harvest.

Glycolytic changes, however, were not restricted to the first 4 days of storage. Significant alterations in enzyme activities and metabolite concentrations also occurred at 7, 10, 30, and 60 DAH. Since wound-healing continues through at least the first 2 weeks in storage (Ibrahim et al., 2001; Fugate et al., 2016), glycolytic changes occurring 7–10 d after storage may have occurred to meet the energy and substrate requirements of wound-healing processes (Gaupels et al., 2012). The cause of glycolytic changes that occurred after longer durations of storage, i.e., after 30 and 60 days storage, when root respiration rate was low and relatively constant and wound-healing was presumably complete, however, are unknown.

The changes in glycolytic enzyme activities during storage exhibited many similarities with the changes in root respiration rate, suggesting a general relationship between the capacity of glycolytic enzymes and respiration rate. When root respiration rate was high (1–4 DAH), 10 of 14 glycolytic enzyme activities were elevated, although elevations in activity were statistically significant for only 7 of the 10 enzymes. When root respiration rate declined (4–7 DAH), six glycolytic enzyme activities also declined. Between 7 and 60 DAH, root respiration rate was statistically unchanged. During this same period, nine glycolytic enzyme activities were statistically unchanged. The observed similarities between glycolytic enzyme activities and respiration rate are not unique to stored sugarbeet root. Such similarities have been reported for other plant products including carrot roots, potato tubers, and tomato and banana fruit (Adams and Rowan, 1970; Beaudry et al., 1989; Trethewey et al., 1998; Obiadalla-Ali et al., 2004).

Control of plant glycolysis is thought to reside largely in the reactions catalyzed by PK and PFK (Givan, 1999; Plaxton and Podestá, 2006). PK, via its ability to catalyze the terminal reaction of glycolysis and influence the concentration of its substrate, PEP, has been implicated as the principal controller of the pathway in plants. Secondary control of glycolysis is believed to reside in PFK, an enzyme strongly inhibited by PEP and therefore regulated by PK activity (Givan, 1999; Plaxton and Podestá, 2006). In stored sugarbeet roots, these enzymes are also likely to have prominent roles in the regulation of glycolysis, and possibly respiration rate.

Similarities between changes in PK activity and respiration rate were obvious in this study. PK activity and respiration rate were elevated during the first 4 days in storage, declined between 4 and 7 days in storage, and remained relatively constant for the remainder of the storage period (**Figures 1**, **2**). Correlation analysis (**Table 2**) and PCA (**Figure 3**) confirmed the similarities between PK activity and respiration rate. In postharvest sugarbeet roots, in which there is minimal engagement of the oxidative pentose phosphate pathway (Wang and Barbour, 1961) and no drain of carbon compounds from glycolysis to support growth, similarity between PK activity and respiration rate suggests that downstream TCA cycle reactions do not limit the availability of substrates for respiration. Changes in the concentration of PEP, the substrate of PK reaction, also bore similarities to respiration rate. PEP concentration increased during the first 4 days in storage, declined between days 4 and 7, and was relatively constant for the remainder of the storage period. The increase in PEP concentration during the first 4 days of storage indicated greater synthesis than catabolism of this compound, consistent with a restriction in glycolytic flux by PK when high respiration rates created a large demand for glycolytic products.

While similarities between PK activity and respiration rate suggest a role for PK in the regulation of glycolysis, concentration changes observed for F6P, G6P, and F1,6P indicate that PFK is also likely to contribute to the regulation of glycolysis in stored sugarbeet roots. During storage, concentrations of F6P and G6P increased, indicating greater synthesis than catabolism of these compounds. These compounds are generally considered to be in equilibrium with each other through the action of the highly active enzyme, G6PI (**Table 1**; Dennis and Blakeley, 2000) and proceed through glycolysis via conversion of G6P to F6P and conversion of F6P to F1,6P. F1,6P, generally decreased during storage, indicating its synthesis did not keep pace with its catabolism. These concentration changes suggest a restriction in the conversion of F6P to F1,6P, a reaction catalyzed by PFK or PFP. Although either enzyme may be limiting the conversion of F6P to F1,6P, PFK is primarily responsible for this reaction in most plant cells (Dennis and Blakeley, 2000). Moreover, PFK activity was sixfold greater, on average, than PFP activity (**Table 1**) in stored sugarbeet roots. Correlation and principal component analysis revealed a positive relationship between PFK activity and PEP concentration (**Table 5** and **Figure 6**). Such a relationship is consistent with regulation occurring primarily at the reactions catalyzed by PFK and PK without a major restriction of glycolysis by the enzymes intermediate of the PFK and PK reactions.

Although major roles for PK and PFK in the regulation of glycolysis in stored sugarbeet roots are indicated by this study, other glycolytic enzymes activities are also likely to contribute to some extent to glycolytic regulation. In addition to the changes in PK and PFK activities, activity changes were noted for HK, FK, UDPase, G6PI, PGM, PFP, TPI, PGK, and PGlyM. The significance of these changes for overall glycolytic flux is unknown. However, where metabolic control analysis has been used to examine regulation of enzymatic pathways, all, or nearly all, enzymes in a pathway contribute to its regulation, with each enzyme exerting differing magnitudes of relative control on the pathway (Moreno-Sánchez et al., 2008).

Sucrose content decreased by approximately 3–4% in the 60 days after harvest under the favorable storage conditions used in this study. The quantity of sucrose lost in this study was similar to that reported by Beaudry et al. (2011) who observed a 3–9% decline in sucrose content for roots stored for 100 days under ideal conditions. Although a decline in sucrose of this magnitude may not be important for many crop or horticultural products, such a loss is economically important for a crop that is grown solely for the sucrose contained within. Many plant products lose carbohydrates in storage, but remain marketable. Any sucrose lost during the storage of sugarbeet roots, however, is a direct loss of the commercial product.

Cellular energy status, as indicated by the ratio of ATP:ADP, declined during storage and was reduced by 66% after 60 days storage (**Figure 4**). The decline in the ATP:ADP ratio was due to a decline in ATP concentration, indicating that oxidative phosphorylation did not keep pace with ATP usage. The ADP concentration did not significantly change during storage. Similar declines in ATP and cellular energy status have been observed in other postharvest products including litchi and longan fruits, with the declines in ATP and energy status related to senescence and the development of postharvest diseases (Su et al., 2005; Wang et al., 2013). For sugarbeet roots, it is unlikely that the decline in ATP concentration and energy status is related to senescence since the taproot serves as an overwintering survival mechanism for the biennial sugarbeet plant. However, susceptibility of sugarbeet roots to storage diseases increases with storage duration (Huijbregts et al., 2013), and enhanced disease susceptibility may relate to the decline in cellular energy status during storage. It was also observed that cellular energy status, ATP concentration, and ADP concentration were unrelated to root respiration rate throughout 60 days storage. Although energy status and adenylate concentrations have been indicated in the regulation of respiration rate in other plant species and tissues (Loef et al., 2001; Covey-Crump et al., 2007), they are unlikely to have a major regulatory effect on sugarbeet root storage respiration rate, a conclusion supported by earlier research (Klotz et al., 2008).

Cellular redox status was affected by storage duration. The ratio of NADH:NAD^+^ declined by as much as 75% during the first 4 days in storage and then recovered by 7 DAH to a level that was statistically similar to that at harvest (**Figure 4**). The decline in the NADH:NAD^+^ ratio was primarily due to a decline in NADH concentration which was depressed during the first 30 days of storage. Respiration was negatively correlated with the NADH:NAD^+^ ratio and positively correlated with NAD^+^ concentration (**Table 4**). Although the significance of these relationships is unknown, they may reflect the status of NADH and NAD^+^ as substrate and product, respectively, of the electron transport chain, which catalyzes the final steps in the respiratory process (Siedow and Day, 2000; Schertl and Braun, 2014). A positive relationship between respiration rate and NAD^+^ has previously been reported in the mitochondria of developing sugarbeet root (Shugaev, 2001). While this study concluded that NAD^+^ concentration restricted respiration rate in developing sugarbeet roots (Shugaev, 2001), NAD^+^ concentration is unlikely to limit respiration in postharvest sugarbeet roots since elevations in NAD^+^ content did not accompany increases in respiration rate. In contrast to the present study and that of Shugaev (2001), root respiration rate was positively related to cellular redox status in sugarbeet roots whose respiration rates were elevated by wounding (Lafta and Fugate, 2011).

Correlation and principal component analysis revealed additional relationships between glycolytic enzyme activities in addition to those discussed above (**Table 2** and **Figure 3**). Of note among these relationships was a positive correlation of FK activity with HK and PFK activities. These enzyme activities also exhibited similar changes in sugarbeet roots in response to injury (Klotz et al., 2006). The similarities in activity changes for FK with HK and PFK suggest that protein levels for these enzymes may be similarly regulated.

Correlation and principal component analysis also identified additional relationships between metabolite concentrations, cellular energy status, and respiration rate (**Table 4** and **Figure 5**). The ratio of ATP:ADP was negatively correlated with F6P concentration, a relationship that may reflect similar changes between the products of FK activity (F6P and ADP) or an inverse relationship between a substrate (ATP) and product (F6P) of FK reaction. PCA analysis identified similarities in G6P and G1P concentration changes, suggesting an equilibrium between these two metabolites via the activity of phosphoglucomutase, an abundant enzyme in sugarbeet roots (**Table 1**). Such an equilibrium between G6P and G1P has been reported in other plant species (Dennis and Blakeley, 2000). PEP and ATP concentration changes were also similar, perhaps due to the ability of ATP to inhibit PK activity as demonstrated for other plant PKs (Ambasht and Kayastha, 2002). It was also observed that root respiration rate was positively correlated with sucrose concentration and negatively correlated with glucose concentration. While these correlations were statistically significant (**Table 4**), indicating similarities in how these parameters changed with respect to time, the importance of these correlations is questionable since most changes in sucrose and glucose concentrations during storage were non-significant (**Figure 4**).

## Conclusion

Profiles of glycolytic enzymes and metabolites revealed numerous changes in glycolytic enzyme capacities and metabolite concentrations throughout 60 days storage. Glycolysis in postharvest sugarbeet roots, therefore, is dynamic and responds not only to the wound-healing and primary carbon needs of freshly harvested roots, but also changes during long-term storage when respiratory demands are low and stable, and roots display no visible signs of stress or physiological change. Overall, a general relationship between root respiration rate and glycolytic enzyme capacities was observed, suggesting that glycolysis restricts respiration, responds to respiratory substrate demand, or is regulated similarly to respiration rate. Major roles for PK and PFK activities in the regulation of postharvest sugarbeet root glycolysis were indicated; other glycolytic enzymes are likely to contribute to the regulation of glycolysis, but to a lesser extent than PK or PFK. Cellular energy status, measured as the ratio of ATP:ADP, declined with storage duration, but bore no relationship to respiration rate, providing further evidence that cellular energy status does not regulate respiration rate in sugarbeet root. Cellular redox status, represented by the ratio of NADH:NAD^+^, was inversely related to respiration rate. The significance of this relationship is unknown, but may reflect of the status of NADH and NAD^+^ as substrate and product, respectively, of respiration.

## Author Contributions

CM, KF, and FF conceived and planned research. CM carried out all enzymatic analyses. CM, AL, and JF carried out metabolite analyses. CM and KF drafted the manuscript. LC assisted with statistical analyses. FF, ED, and EL provided technical assistance and assisted with manuscript revision.

## Conflict of Interest Statement

The authors declare that the research was conducted in the absence of any commercial or financial relationships that could be construed as a potential conflict of interest.
