# Supplementary material for: Glycolysis Is Dynamic and Relates Closely to Respiration Rate in Stored Sugarbeet Roots
Source: Front Plant Sci. 2017 May 24;8:861. doi: 10.3389/fpls.2017.00861 (PMC5442176; doi:10.3389/fpls.2017.00861)
Supplement: Supplementary file 1 [file Table_1.DOCX]

### SUPPLEMENTARY TABLE S1: ENZYME ASSAY PROTOCOLS

All assays were carried out in 96 well flat bottom microplates at 25°C. Activity was determined by the change in absorbance at 340 nm using a SpectraMAX Plus microplate spectrophotometer (Molecular Devices Corp., Sunnyvale, CA, USA). Asterisks denote reagents that were used to start reactions. The extinction coefficient for NADH (6220 M^-1^ cm^-1^) was used to quantify enzyme activity.

| **Enzyme** | **Solution I** | **Solution II** | **Solution III** | **Assay solution** | **Assay** |
| --- | --- | --- | --- | --- | --- |
| **Hexokinase**  **(HK)** | 250 mM HEPES – pH  7.5  20 mM MgCl_2_  14 mM glucose | 75 mM NAD | 2 U µl^-1^ glucose 6-phosphate dehydrogenase | 1000 μL Sol’n I  40 μL Sol’n II  2 μL Sol’n III | 90 µL assay solution  60 µL extract  25 µL water  5 µL 50 mM ATP * |
| **Fructokinase**  **(FK)** | 250 mM HEPES – pH  7.5  20 mM MgCl_2_  6 mM fructose | 75 mM NAD | 1) 2 U µl^-1^ glucose 6-phosphate dehydrogenase  2) 6 U µl^-1^ phospho- glucose isomerase | 1000 μL Sol’n I  40 μL Sol’n II  Sol’n III (2 μL #1, and 2 μL #2) | 90 µL assay solution  60 µL extract  25 µL water  5 µL 50 mM ATP* |
| **Phosphofructokinase**  **(PFK)** | 100 mM TRIS-HCl–pH  8.0  20 mM MgCl_2_  2 mM EDTA  2 mM fructose 6-phosphate | 40 mM NADH | 1) 2 U µl^-1^ aldolase  2) 2 U µl^-1^ triose phosphate isomerase  3) 5 U µl^-1^ glycerol 3-phosphate dehydrogenase | 1000 μL Sol’n I  5 μL Sol’n II  Sol’n III (2 μL #1, 2 μL #2 and 2 μL #3) | 90 µL assay solution  60 µL extract (5 x dilution)  25 µL water  5 µL 24 mM ATP* |
| **Pyruvate kinase**  **(PK)** | 100 mM HEPES – pH  7.0  100 mM KCl  20 mM MgCl_2_  4 mM DTT  BSA (0.4 mg/mL) | 1) 15 mM NADH  2) 200 mM PEP | 26 U µl^-1^ lactate dehydrogenase | 1000 μL Sol’n I  Sol’n II (10 μL#1 and 10 μL #2)  2 μL Sol’n III | 90 µL assay solution  20 µL extract (5 x dilution)  50 µL water  20 µL 50 mM ADP* |
| **Phosphoglucomutase**  **(PGM)** | 100 mM TRIS-HCl–pH  7.5  20 mM MgCl_2_ | 1) 100 mM NAD^+^  2) 30 µM glucose 1,6-P_2_ | 2 U µl^-1^ glucose 6-phosphate dehydrogenase | 1000 μL Sol’n I  Sol’n II (10 μL #1 and 0.9 μL #2)  2 μL Sol’n III | 90 µL assay solution  15 µL extract (5 x dilution)  65 µL water  10 µL 90 mM glucose 1-phosphate* |
| **Glucose 6-phosphate isomerase**  **(G6PI)** | 75 mM Gly-Gly – pH 8.5  10 mM MgCl_2_ | 1) 150 mM NAD^+^  2) 150 mM fructose 6-phosphate | 0.5 U µl^-1^ glucose 6-phosphate dehydrogenase | 1000 μL Sol’n I  Sol’n II (10 μL #1 and 10 μL #2)  2 μL Sol’n III | 120 µL assay solution  50 µL water  10 µL extract (10 x dilution)* |
| **Aldolase**  **(ALD)** | 40 mM HEPES – pH 7.7 | 1) 15 mM NADH  2) 300 mM fructose 1,6-P_2_ | 1) 1.7 U µl^-1^ glycerol 3-phosphate dehydrogenase  2) 17 U µl^-1^ triose phosphate isomerase | 1000 μL Sol’n I  Sol’n II (10 μL #1 and 25 μL #2)  Sol’n III (2 μL #1 and 2 μL #2) | 120 µL assay solution  40 µL water  20 µL extract (5 x dilution)* |
| **Triose phosphate isomerase**  **(TPI)** | 100 mM HEPES – pH  8.0  1.5 mM DL-glyceraldehyde 3-phosphate  5 mM EDTA | 30 mM NADH | 1 U µl^-1^ glycerol 3-phosphate dehydrogenase | 1000 μL Sol’n I  10 μL Sol’n II  2 μL Sol’n III | 120 µL assay solution  40 µL water  20 µL extract (200 x dilution)* |
| **Glyceraldehyde**  **3-phosphate**  **dehydrogenase**  **(GAPDH)** | 100 mM TRIS – pH 7.8  4.5 mM 3-phosphoglycerate  8 mM MgSO_4_  1 mM EDTA  2 mM DTT | 1) 50 mM NADH  2) 300 mM ATP | 1.8 U µl^-1^ 3-phosphoglycerate kinase | 1000 μL Sol’n I  Sol’n II (10 μL #1 and 10 μL #2)  2 μL Sol’n III | 120 µL assay solution  20 µL water  40 µL extract (2 x dilution)* |
| **Phosphoglycerate kinase**  **(PGK)** | 100 mM HEPES – pH  7.6  1 mM EDTA  2 mM MgSO_4_  6.5 mM 3-  phosphoglycerate | 1) 25 mM NADH  2) 110 mM ATP | 3.3 U µl^-1^ glycerol 3-phosphate dehydrogenase | 1000 μL Sol’n I  Sol’n II (10 μL #1 and 10 μL #2)  2 μL Sol’n III | 165 µL assay solution  15 µL extract* |
| **Phosphoglycerate mutase**  **(PGlyM)** | 100 mM TRIS – pH 7.6  10 mM MgSO_4_  4 mM DTT  BSA (0.4 mg/mL) | 1) 75 mM NADH  2) 250 mM ADP | 1) 1 U µl^-1^ enolase  2) 5 U µl^-1^ pyruvate kinase  3) 6 U µl^-1^ lactate dehydrogenase | 1000 μL Sol’n I  Sol’n II (5 μL #1 and 20 μL #2)  Sol’n III (2 μL #1, 2 μL #2 and 2 μL #3) | 100 µL assay solution  30 µL extract  19 µL water  11 µL 50 mM 3-phosphoglycerate* |
| **Enolase**  **(ENO)** | 100 mM HEPES – pH  7.5  10 mM MgCl_2_ | 1) 180 mM NADH  2) 250 mM ADP | 1) 5 U µl^-1^ pyruvate kinase  2) 6 U µl^-1^ lactate dehydrogenase | 1000 μL Sol’n I  Sol’n II (10 μL #1 and 20 μL #2)  Sol’n III (2 μL #1 and 2 μL #2) | 100 µL assay solution  15 µL extract  56 µL water  9 µL 10 mM 2-phosphoglycerate* |
| **Phosphoenolpyruvate phosphatase**  **(PEPase)** | 50 mM TRIS-HCl – pH  7.5  4 mM MgCl_2_ | 1) 30 mM NADH  2) 150 mM PEP | 3 U µl^-1^ lactate dehydrogenase | 1000 μL Sol’n I  Sol’n II (10 μL #1 and 10 μL #2)  2 μL Sol’n III | 120 µL assay solution  40 µL water  20 µL extract* |
| **Pyrophosphate-dependent phosphofructokinase**  **(PFP)** | 100 mM TRIS – pH 8.0  5 mM fructose 6-phosphate  5 mM MgCl_2_  2 mM PP_i_ | 40 mM NADH | 1) 1 U µl^-1^ aldolase  2) 1.3 U µl^-1^ glycerol 3-phosphate dehydrogenase  3) 10 U µl^-1^ triose phosphate isomerase | 1000 μL Sol’n I  10 μL Sol’n II  Sol’n III (2 μL #1, 2 μL #2 and 2 μL #3) | 100 µL assay solution  50 µL water  30 µL extract (200 x dilution)* |
| **UDP-glucose pyrophosphorylase**  **(UDPase)** | 100 mM TRIS – pH 8.0  5 mM MgCl_2_  0.8 mM UDP-glucose | 85 mM NAD^+^ | 1) 4 U µl^-1^ phospho- glucomutase  2) 4 U µl^-1^ glucose 6-phosphate isomerase | 1000 μL Sol’n I  20 μL Sol’n II  Sol’n III (2 μL #1 and 2 μL #2) | 172 µL assay solution  6 µL extract  2 µL 200 mM sodium pyrophosphate* |
