# Supplementary material for: Glycolysis Is Dynamic and Relates Closely to Respiration Rate in Stored Sugarbeet Roots
Source: Front Plant Sci. 2017 May 24;8:861. doi: 10.3389/fpls.2017.00861 (PMC5442176; doi:10.3389/fpls.2017.00861)
Supplement: Supplementary file 2 [file Table_2.DOCX]

**SUPPLEMENTARY TABLE S2: METABOLITE QUANTIFICATION PROTOCOLS**

**Sucrose, glucose, and fructose:**

End point colorimetric reactions were used to determine concentrations of glucose, glucose and fructose, and sucrose and glucose from which the individual concentrations of glucose, fructose, and sucrose were calculated. Reactions were conducted in flat-bottom 96 well plates with all reaction components pipetted into wells using an automated liquid handler (Janus Varispan Automated Workstation, Perkin Elmer, Shelton, CT). Assays for glucose and the combined concentration of glucose and fructose, contained 5 μL extract and 195 μL of Glucose UV liquid reagent (GlcRgt; Cliniqa, San Marcos, CA, USA) or GlcRgt + phosphoglucose isomerase (1470 U L^-1^), respectively. The combined concentration of sucrose and glucose was determined by incubating 10 μL extract with 90 μL invertase solution (17,500 U L^-1^ invertase in 30 mM sodium acetate, pH 4.6) for 10 min at 25^o^C, followed by incubation of a 5 μL aliquot of this solution with 195 μL GlcRgt. Microplates were centrifuged for 1 minute at 800 x g and 4^o^C after solutions were added, and reactants were mixed by agitation for 1 minute. Reactions were incubated at 25^o^C for 10 minutes and absorbance was measured at 340 nm with a SpectraMax Plus microplate spectrophotometer (Molecular Devices, Sunnyvale, CA). Carbohydrate concentrations were determined against standard curves. Concentrations of fructose and sucrose were determined by subtraction of glucose concentration from the values obtained for the combined concentration of glucose and fructose and the combined concentration of sucrose and glucose.

**Glucose 6-phosphate, fructose 6-phosphate, and glucose 1-phosphate**

End point colorimetric reactions were used to determine concentrations of glucose 6-phosphate, fructose 6-phosphate, and glucose 1-phosphate. Tissue extract was added to a solution containing 100 mM Tris-HCl (pH 8.1), 5 mM MgCl_2_, and 0.25 mM NADP^+^ in a total volume of 175 μL. Sequential additions of 0.2 U glucose-6-phosphate dehydrogenase (G6PDH), 0.2 U phosphoglucose isomerase, and 0.06 U phosphoglucomutase were made at 25 ^o^C at 2 minute intervals with absorbance at 340 nm determined after each addition with a SpectraMAX Plus microplate reader. Concentrations were determined against standard curves.

**Triose phosphate and fructose 1,6-bisphosphate**

Triose phosphate and fructose 1,6-bisphosphate concentrations were determined as described above for glucose 6-phosphate, fructose 6-phosphate and glucose 1-phosphate except NADP^+^ was replaced by 150 μM NADH and absorbance changes were determined after sequential additions of 0.25 U glycerophosphate dehydrogenase, 0.6 U triose phosphate isomerase, and 0.05 U aldolase.

**NAD^+^ and NADH**

NAD^+^ and NADH concentrations were determined by the change in absorbance at 570 nm after a 30 minute, 37 ^o^C incubation of a solution containing 100 mM bicine-NaOH buffer (pH 8.0), 4 mM EDTA, 0.42 mM 3-(4,5-dimethylthiazol-2-yl)-2,5-diphenyl-tetrazolium bromide (MTT), 1.7 mM phenazine ethosulfate (PES), 500 mM ethanol, 2 U alcohol dehydrogenase (ADH), and extract in a total volume of 200 μL. Reactions were started by the addition of ADH. NAD^+^ and NADH concentrations were determined against standard curves.

**Phosphoenolpyruvate, pyruvate, ADP, and ATP**

Phosphoenolpyruvate, pyruvate, ADP, and ATP concentrations were determined after 10-fold dilution of extracts and injection onto a 250 x 4.6 mm Prevail organic acid column (Grace Davison Discovery Science, Deerfield, IL, USA). Compounds were eluted with 25 mM KH_2_PO_4_ (pH 2.5) at 1 mL min^-1^, and detected spectroscopically at 210 nm. External standards and calibration curves were used to identify and quantify metabolites.

**UDP and UDP-glucose**

UDP and UDP-glucose concentrations were quantified spectroscopically at 254 nm after extracts were passed over a 250 x 4.6 mm Partisil SAX anion-exchange column (Alltech Associates, Deerfield, IL, USA). Compounds were eluted at 1 mL min^-1^ using a gradient comprised of (A) 10 mM NH_4_H_2_PO_4_ (pH 2.8) and (B) 750 mM NH_4_H_2_PO_4_ (pH 3.7), in which component B was held at 7.5% for 12 minutes, increased to 90% over 3 minutes, increased to 100% over 10 minutes and held at 100% for an additional 7 minutes. External standards and calibration curves were used to identify and quantify metabolites.
