# Supplementary material for: Glycolysis Is Dynamic and Relates Closely to Respiration Rate in Stored Sugarbeet Roots
Source: Front Plant Sci. 2017 May 24;8:861. doi: 10.3389/fpls.2017.00861 (PMC5442176; doi:10.3389/fpls.2017.00861)
Supplement: Supplementary file 8 [file Image_3.PDF]

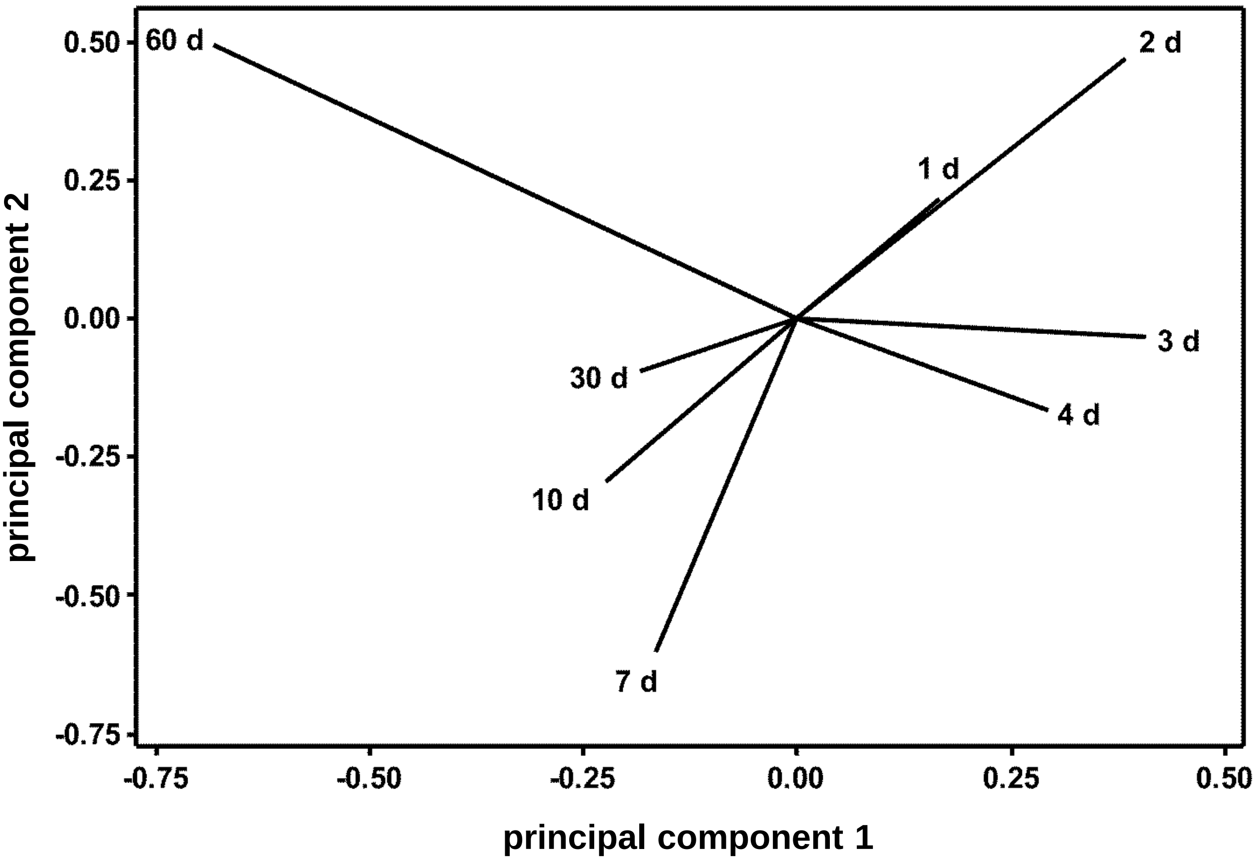

FIGURE S3 | Principal component analysis (PCA) loading plot of the changes in glycolytic enzyme activities, metabolite concentrations, ATP:ADP ratio, NADH:NAD^+ ratio, and respiration rate (RESP) during 60 d storage. Roots were stored 10 d at 10 °C and 90 ± 5% relative humidity (RH), then stored at 4 °C, 90 ± 5% RH for 50 d. Principal component 1 and principal component 2 account for 34 and 26%, respectively, of the variance in the data after standardization.
